# Change in Plasma Biomarkers of Alzheimer’s Disease From Midlife to Late-Life and Associations With Cognitive Change

**DOI:** 10.1093/geroni/igaf122.209

**Published:** 2025-12-31

**Authors:** Priya Palta, James Pike, Keenan Walker, Kevin Sullivan, Bharat Thyagarajan, Thomas Mosley, Josef Coresh

**Affiliations:** UNC School of Medicine, Chapel Hill, North Carolina, United States; New York University, New York, New York, United States; National Institute on Aging, Baltimore, Maryland, United States; University of Mississippi Medical Center - The MIND Center, Jackson, Mississippi, United States; University of Minnesota, Minneapolis, Minnesota, United States; University of Mississippi Medical Center - The MIND Center, Jackson, Mississippi, United States; New York University, New York, New York, United States

## Abstract

Plasma biomarkers of Alzheimer’s disease (AD) pathology offer a non-invasive method to assess brain changes linked to dementia and cognitive decline. However, the associations with specific cognitive domains and subsequent cognitive change in participants with or without cognitive impairment is unclear. Plasma biomarkers were assayed in ARIC participants using Quanterix SiMoA technology on samples from midlife (visit 3, 1993-95, mean age 59) and late-life (visit 5, 2011-13, mean age 77). Biomarkers included Aβ42:Aβ40 ratio, p-Tau181, neurofilament light (NfL), and GFAP. Global and domain-specific cognitive factor scores were calculated from neuropsychological tests administered across multiple visits. Linear mixed effects models estimated the association between biomarkers and late-life cognitive change, adjusting for demographics, lifestyle and cardiovascular risk factors, and APOE-ε4 status. Exploratory analyses examined effect modification of the biomarker-global cognition associations by visit 5 cognitive impairment. Among 1515 participants with plasma biomarkers (60.7% female, 25.5% identified as Black), 39% had cognitive impairment in late-life. Higher midlife NfL and GFAP were associated with faster declines in global cognition and language. NfL also predicted faster memory decline. In late-life, lower Aβ42:Aβ40 and higher p-Tau181, NfL, and GFAP were associated with faster declines in global cognition, memory, and language. Changes in NfL, p-Tau181, and GFAP from midlife to late-life were associated with declines in global cognition and language. No consistent effect modification by cognitive impairment was observed. Plasma biomarkers of AD pathology, neuronal injury, and astrogliosis show distinct associations with cognitive decline across domains when measured in midlife and late-life.

